# Cost-effectiveness of screening for anal cancer using regular digital ano-rectal examinations in men who have sex with men living with HIV

**DOI:** 10.7448/IAS.19.1.20514

**Published:** 2016-03-01

**Authors:** Jason J Ong, Christopher K Fairley, Susan Carroll, Sandra Walker, Marcus Chen, Tim Read, Andrew Grulich, Catriona Bradshaw, John Kaldor, Philip Clarke

**Affiliations:** 1 Sexual Health Unit, Melbourne School of Population and Global Health, University of Melbourne, Melbourne, Australia; 2 Melbourne Sexual Health Centre, Alfred Health, Carlton, Australia; 3 Department of Sexual Health, Central Clinical School, Monash University, Clayton, Australia; 4 Chris O'Brien Lifehouse, Camperdown, Australia; 5 Department of Medicine, Kirby Institute, University of New South Wales, Darlinghurst, Australia

**Keywords:** HIV, anal cancer, men who have sex with men, cost-effectiveness, screening

## Abstract

**Introduction:**

Anal cancer in men who have sex with men (MSM) living with HIV is an important issue but there are no consistent guidelines for how to screen for this cancer. In settings where screening with anal cytology is unavailable, regular anal examinations have been proposed in some guidelines but their cost-effectiveness is unknown.

**Methods:**

Our objective was to estimate the cost-effectiveness of regular anal examinations to screen for anal cancer in HIV-positive MSM living in Australia using a probabilistic Markov model. Data sources were based on the medical literature and a clinical trial of HIV-positive MSM receiving an annual anal examination in Australia. The main outcome measures for calculating effectiveness were undiscounted and discounted (at 3%) lifetime costs, life years gained, quality-adjusted life years (QALY) gained and incremental cost-effectiveness ratio (ICER).

**Results:**

Base-case analysis estimated the average cost of screening for and management of anal cancer ranged from $195 for no screening to $1,915 for lifetime annual screening of men aged ≥ 50. Screening of men aged ≥ 50 generated ICERs of $29,760 per QALY gained (for screening every four years), $32,222 (every three years) and $45,484 (every two years). Uncertainty for ICERs was mostly influenced by the cost (financially and decrease in quality of life) from a false-positive result, progression rate of anal cancer, specificity of the anal examination, the probability of detection outside a screening program and the discount rate.

**Conclusions:**

Screening for anal cancer by incorporating regular anal examinations into routine HIV care for MSM aged ≥ 50 is most likely to be cost-effective by conventional standards. Given that anal pap smears are not widely available yet in many clinical settings, regular anal exams for MSM living with HIV to detect anal cancer earlier should be implemented.

## Introduction

Anal cancer occurs commonly in men who have sex with men (MSM) living with HIV (65 to 109 per 100,000 person-years) [1] than the general population (incidence 1 to 2 per 100,000 person-years) [2]. In response to these high rates, screening strategies are being investigated. One uses a secondary prevention model to detect the precursor lesion (high-grade squamous intra-epithelial lesions, HSIL) using anal pap smears to identify HSIL, so that treatment may prevent progression to anal cancer [3]. Implementation of this method is hampered by a lack of trained high-resolution anoscopists and insufficient evidence that it reduces anal cancer morbidity or mortality [4]. Furthermore cost-effectiveness analyses for this intervention have been conflicting, with a US study finding it cost-effective [5] but a UK study found that it was not cost-effective regardless of the frequency of screening [6,7]. A second approach uses a tertiary prevention model of regular anal examinations for early cancer detection although there is limited evidence for this as well. Only two HIV guidelines advocate for regular anal cancer examinations (ACEs) in those living with HIV based only on evidence from “expert opinion” [8] There are no published studies to support the economic basis for the recommendation and no randomized controlled trials that demonstrate the efficacy of digital ano-rectal examinations (DARE). However, anal cancers are diagnosed late when the prognosis is substantially worse [9]. Furthermore, if perianal cancers could be detected at a size less than 2 cm, curative local excision may be possible [10] and thereby avoid combined chemoradiation, which is the mainstay of treatment.

Several factors favour the incorporation of regular DARE into routine HIV care. Firstly, most HIV patients in high-income countries generally see their HIV doctor at least annually for HIV care. Secondly, anal cancers are diagnosed late and most anal cancers are either visible by inspection of the peri-anal area or palpable by DARE [11]. Thirdly, regular DARE in MSM living with HIV found them to be highly acceptable and did not result in a large health care expenditure from non-cancer findings [12]. In order to assist with a decision about implementing DARE based on the best available evidence, we have undertaken a Markov decision model [13]. Cost-effectiveness studies evaluating anal cancer screening using anal cytology have also used Markov modelling [5,6] but this is the first cost-effectiveness evaluation of an early cancer detection intervention using regular DARE, as opposed to an intervention aimed at detection and treatment of cancer precursors.

The objective of our study was to estimate the cost-effectiveness of regular anal examinations to screen for anal cancer in HIV-positive MSM living in Australia. The findings of this article will provide evidence for policy makers to consider when implementing anal cancer screening for HIV-positive MSM.

## Methods

### Clinical data

We chose to restrict our model to screen for MSM living with HIV because they have the highest incidence for anal cancer (78 per 100,000 person-years) compared with MSM without HIV (5 per 100,000 person-years) [1]. It is unlikely that offering screening to MSM without HIV would be cost-effective given their much lower incidence for anal cancer. Our analysis used published and clinical data from the ACE study, a prospective cohort study of 327 MSM living with HIV in Melbourne, Australia, where men received an annual DARE over two years [14]. The clinical study provided information on patient uptake of screening, costs of screening (including extra consultation time, complication rates from screening) and utility weights for calculating quality-adjusted life years (QALYs). This research was approved by the Alfred Health Human Ethics Committee (Project 246/12).

### Model structure (Supplementary file 1)

The Markov model was similar to one used for colorectal cancer screening [15]. Supplementary file 1 shows the structure of our model for the intervention arm. The comparator arm has the exact structure with the probabilities of detection through screening set to 0 in the relevant arms. At the start of the model, a 35-year-old MSM living with HIV had no cancer but could transition into one of the following health states: localized cancer (detected/undetected), regional cancer (detected/undetected), distal cancer (detected/undetected), remission from anal cancer and death. Transition probabilities were estimated from the literature and are reported in Table 1 and Supplementary files 3 and 4. The model progresses in cycles of one year with a time horizon of a maximum age of 100 years.

**Table 1:** Base-case values and ranges used in sensitivity analysis

Variable	Value (Range)	References
Specificity of DARE*	0.25 (0.1 to 0.5)	12
Sensitivity of DARE to detect a cancer <2 cm	0.9 (0.70 to 1.00)	Assumption
Mean probability of developing anal cancer	Age-specific	[16], Supplementary file 3
Probability of localized anal cancer diagnosed not because of screening	0.2 (0.1 to 0.55)	[5]
Probability of dying from not-related to anal cancer	Age-specific	[17]
Probability of survival from anal cancer	Stage-specific	[16], Supplementary file 4
Costs ($AUD)		
Screening	16 (10 to 30)	[18–20]
False-positive	218 (100 to 500)	[14,18–20]
Workup	1,864 (1,000 to 3,000)	[10,18–20]
Localized cancer treatment	10,386 (5,000 to 15,000)	[10,18–20]
Regionalized cancer treatment	11,093 (5,000 to 15,000)	[10,18–20]
Distal cancer treatment	14,638 (10,000 to 20,000)	[10,18–20]
Monitoring	Variable according to time from diagnosis	[10,18–20]
Utility weights		
No cancer	0.76 (0.66 to 0.86)	[14]
Localized cancer	0.71 (0.56 to 0.76)	Assumption
Regional cancer	0.66 (0.56 to 0.76)	[21]
Distal cancer	0.52 (0.42 to 0.62)	[21]

* Specificity refers to the proportion of cases referred to a colorectal surgeon that resulted in a diagnosis of anal cancer.

The model evaluated the screening intervention as an anal examination (estimated to take an extra five minutes) conducted by a trained clinician as part of a regular HIV consultation. If a suspicious lesion was identified, the patient would be referred to a colorectal surgeon for further evaluation. The strategies assessed included conducting DARE yearly, every two years, every three years, every four years and every five years. Age for initiating screening was also varied (aged 35 vs. 50 years). A starting age of 35 years was initially chosen because it is rare to have anal cancer detected below this age [2]. We also examined a policy of initiation at 50 years of age, as the anal cancer incidence rate is almost nine times greater for those aged 50 to 64 compared with those aged 35 to 50 years [2]. The main outcomes of the model were (i) quality-adjusted life years (QALY) to facilitate comparison with several previous studies of interventions involving anal cancer screening [5–7] and because they are commonly used as an outcome for determining government funding (e.g. decision for funding pharmaceuticals in Australia) [22] and (ii) lifetime healthcare costs to the intervention's impact on downstream costs to be evaluated.

### Data inputs

This study employed Australian costs because a detailed costing of anal cancer management could be conducted. We had access to detailed country level administrative information on the costs and procedures undertaken in detecting and managing anal cancer. Costs for other countries are considered in the sensitivity analysis.

We chose the perspective of a government as the primary third-party funder and costs (in Australian dollars, 2014) were derived from Medicare [18,23] (Australia's universal public health scheme) and the Australian refined diagnosis-related groups data [19,20] (which provided activity-based hospital costing) (Supplementary file 2). We verified the codes with clinical coders from hospitals that provided care for anal cancer patients. Costs were those associated with the screening and management of anal cancer. We did not include capital and overhead costs of running the medical facilities (e.g. administrative staff, building costs and utilities) as it is presumed that regardless of whether screening is implemented or not, these men would still attend their standard HIV consultation visit.

We consulted with an Australian radiation oncologist (SC) who treats anal cancer and used the National Comprehensive Cancer Network clinical practice guidelines [10] to determine the pathway of care (i.e. workup, treatment, monitoring) for anal cancer (Supplementary file 2). For management costs, we assumed that HIV patients with anal cancer were given the same regimens as non-HIV patients [24] and had similar outcomes [25]. Costs for monitoring patients after being diagnosed with anal cancer were based on a typical anal cancer patient being treated in a metropolitan Australian city.

Utility weights for all model health states are summarized in Table 1. Our literature review for utility weights only found studies where an overall anal cancer health state was estimated [26] and no studies provided stage-specific utility weights for anal cancer. Consistent with previous anal cancer cost-effectiveness studies [5,6], we used weights derived from colorectal cancer and used the results of a meta-analysis of colorectal cancer utilities as proxy health-related quality of life associated with anal cancer [21]. In the absence of studies on the sensitivity of anal cancer detection using DARE, we assumed that the sensitivity of visualizing a perianal lump or ulcer or feeling an intracranial lump would be 90% (range 70 to 100%). This is a reasonably conservative estimate given that 52% of anal cancers are already visible [11], so that only a further 38% would need to be detected from palpation. This would be much lower than the estimates for feeling a lump through breast tissue (sensitivity of 75% [27]).

The mechanism by which DARE saves years of life and QALYs will be through treatment of detected anal cancers (i.e. by detecting cancer at an earlier stage through a screening programme, treatment would lead to increased QALYs). The standard treatment for anal cancer is chemoradiation unless a perianal cancer is detected early (i.e. <2 cm) whereby surgical excision without chemoradiation is possible [10]. As there were no specific stage-specific survival data for MSM living with HIV, we estimated stage-specific survival using the Surveillance, Epidemiology, and End Results (SEER) programme of the National Cancer Institute in the United States [16] to calculate age-standardized relative survival and age-specific anal cancer incidence using SEER*Stat that reported on anal cancer from 1973 to 2011. This is likely to be a conservative measure as survival from anal cancer is expected to improve with newer treatment modalities [28] Supplementary file 3 summarizes the age-specific incidence for anal cancer. To calculate age-standardized relative survival, we used the data from 2,628 men with localized cancer, 1,595 men with regional cancer and 554 men with distal cancer. We fitted a survival curve to these data and calculated an equation for the annual probability of survival using a Weibull distribution (Supplementary file 4). In our model, those who have been treated for anal cancer do not go back to a cancer-free state but follows these survival curves extrapolated from the SEER data. For those who do not have cancer, their probability of death from other causes was based on data from Australia's National HIV Registry [17] to accurately reflect competing risks in this population.

As it is unethical to observe the natural history of cancer, our literature review did not find any published data on the progression rates of anal cancer from local to regional to metastatic disease. After discussions with the authors of this article (which includes sexual health physicians (CK, MC, TR, CB) and a radiation oncologist (SC) who manages anal cancer in MSM living with HIV), consensus was reached that it is clinically plausible for 100% of undetected anal cancer to progress sequentially from local to regional to distal cancer over one-year periods and that death from anal cancer would only occur after one year after undetected distal cancer. Our model did not allow for a person to skip over this sequence of events but a man can still die from causes other than anal cancer regardless of what stage of cancer they have. Given the uncertainty of the natural history of anal cancer, we included in our sensitivity analyses models where progression rates were over two- and three-year intervals, instead of one year.

### Model validation

Our literature review found that the SEER database provided the most detailed information on anal cancer at a population level. Their summary of the cancer stage at diagnosis for anal cancer – 49% local, 32% regional, 12% distal cancer, 7% unknown [29] – closely matched our model's estimate of 52% local, 31% regional and 17% distal cancer. Furthermore, the SEER database provided a summary of the distribution of anal cancer diagnosed according to age as 1% (aged 20 to 34), 7% (aged 35 to 44), 25% (aged 45 to 54), 28% (aged 55 to 64), 19% (aged 65 to 74), 14% (aged 75 to 84) and 6% (aged >84). This matched our model's estimate in the no screening group of 10% (aged 35 to 44), 22% (aged 45 to 54), 28% (aged 55 to 64), 18% (aged 65 to 74), 14% (aged 75 to 84) and 8% (aged >84). In addition, our model's predicted lifetime risk of anal cancer was 3% and was in keeping with the estimated lifetime risk for anal cancer from a meta-analysis [1].

### Analyses

We used Bayesian revision to calculate post-test probabilities using the pre-test probability of the prevalence of anal cancer and test characteristics of DARE. Costs and QALYs saved were discounted with an annual rate of 3% (with sensitivity analysis of 0 and 5%) and we used half-cycle correction to account for these discounts. The main outcomes identified were undiscounted and discounted lifetime costs, life years (LY) gained, QALYs gained and incremental cost-effectiveness ratio (ICER). We conducted the cost-effectiveness analysis using TreeAge Pro (TreeAge Software Inc., Williamstown, MA). To calculate the ICER, we ranked strategies according to increasing effectiveness (LY or QALY gained) and those that were less effective and more expensive (i.e. dominated) compared with the preceding strategy were removed. Then, strategies exhibiting extended dominance (ICER was higher than a more effective screening strategy) were also removed and final ICERs were calculated between remaining strategies. To calculate the average cost-effectiveness ratio (ACER), we compared each strategy with the base-case (i.e. no screening).

Univariate sensitivity analyses were conducted to assess the stability of the conclusions to plausible changes in uncertain parameters. This is important as this will determine the likely range of ICER given potential variation in key parameters such as discount rate; costs of screening and managing anal cancer; cost of false-positives; uptake of screening; sensitivity and specificity of detecting anal cancers by clinicians; incidence of anal cancer; progression rates of anal cancer; and utility weight depreciation associated with anal cancer stages and its treatment. To address sampling uncertainty and to capture variability around point estimates concurrently, a probabilistic sensitivity analysis was performed using second-order Monte Carlo simulation. This involved randomly selected values from each input parameter's distribution and generated results for that combination of values. This process was repeated 10,000 times. Gamma distributions were used for costs, beta distributions for utilities and transition probabilities. Standard deviations were derived from our clinical study or assumed to be+30% of the input parameter when there were insufficient data.

## Results

### Base-case analysis

The average undiscounted lifetime cost of screening for and managing anal cancer ranged from $373 ($195 at 3% discount) for no screening to $4,468 ($1,915 at 3% discount) for annual screening of men aged ≥50 (Table 2 and Table 3). The average number of undiscounted life years gained ranged from 46.23 (24.20 at 3% discount) for no screening, to 46.39 (24.25 at 3% discount) for annual screening of men aged ≥50. The average number of undiscounted QALYs gained ranged from 35.11 (18.39 at 3% discount) for no screening to 35.24 (18.42 at 3% discount) for annual screening of men aged ≥50. All ten screening strategies for anal cancer increased the number of QALYs gained and were more costly than not screening.

**Table 2:** Base-case analysis of *undiscounted* costs, quality-adjusted life years gained, the average- and incremental cost-effectiveness of lifetime screening strategies for anal cancer for a 35-year-old HIV-positive MSM. (Comparing each screening strategy with the next most cost-effective option)

Screening strategy	Costs ($AUD, 2014)	Life years	ICER ($ per life year gained)	QALY	ICER ($ per QALY gained)	ACER ($ per QALY gained)
No screening	373	46.2328	N/A	35.1146	N/A	N/A
Age 35–49 every five years	755	46.2434	*	35.1225	*	48,354
Age 35–49 every four years	883	46.2487	*	35.1264	*	43,220
Age 35–49 every three years	1,008	46.2528	*	35.1295	*	42,617
Age 35–49 every two years	1,385	46.2642	*	35.1382	*	42,881
Age 35–49 every year	2,250	46.2722	*	35.1445	*	62,776
Age ≥50 every five years	1,279	46.2953	*	35.1607	*	19,653
Age ≥50 every four years	1,495	46.3102	14,496	35.1717	19,650	19,650
Age ≥50 every three years	1,833	46.3304	16,733	35.1877	21,125	19,973
Age ≥50 every two years	2,506	46.3600	22,736	35.2104	29,648	22,265
Age ≥50 every year	4,468	46.3938	58,047	35.2379	71,345	33,212

ICER, incremental cost-effectiveness ratio; ACER, average cost-effectiveness ratio; QALY, quality-adjusted life years.

* Strategy has a higher incremental cost-effectiveness ratio than a more effective alternate strategy.

**Table 3:** Base-case analysis of *discounted* (3%) costs, quality adjusted life years gained, the average- and incremental cost-effectiveness of lifetime screening strategies for anal cancer for a 35-year-old HIV-positive MSM. (Comparing each screening strategy with the next most cost-effective option)

Screening strategy	Costs ($AUD, 2014)	Life years	ICER ($ per life year gained)	QALY	ICER ($ per QALY gained)	ACER ($ per QALY gained)
No screening	195	24.2034	N/A	18.3857	N/A	N/A
Age 35 to 49 every five years	529	24.2091	*	18.3899	*	79,524
Age 35 to 49 every four years	629	24.2118	*	18.3919	*	70,000
Age 35 to 49 every three years	736	24.2139	*	18.3936	*	68,481
Age 35 to 49 every two years	1,033	24.2191	*	18.3977	*	69,833
Age ≥50 every five years	600	24.2217	*	18.3993	*	29,779
Aged 35 to 49 every year	1,745	24.2229	*	18.4008	*	102,649
Age ≥50 every four years	692	24.2259	22,089	18.4024	29,760	29,760
Age ≥50 every three years	837	24.2314	26,364	18.4069	32,222	30,283
Age ≥50 every two years	1,119	24.2394	35,250	18.4131	45,484	33,723
Age ≥50 every year	1,915	24.2483	89,438	18.4206	106,133	49,284

ICER, incremental cost-effectiveness ratio; QALY, quality-adjusted life year.

* Strategy has a higher incremental cost-effectiveness ratio than a more effective alternate strategy.

If we implemented screening for 1,000 men aged ≥50 every four years, 12 out of 21 (57%) of anal cancers detected would be localized; for screening every three years, 14 out of 22 (64%) of anal cancers detected would be localized; for screening every two years, 18 out of 24 (75%) of anal cancers detected would be localized; and for screening every year, 23 out of 26 (88%) of anal cancers detected would be localized.

**Figure 1:**
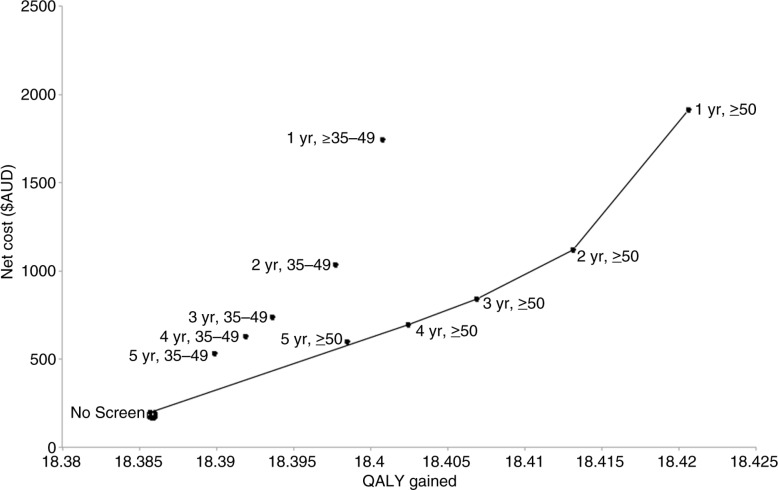
Cost effectiveness plane of anal cancer screening strategies in HIV-positive MSM (base-case). Screening strategies legend: 5 years, 35–49=screening every five years for men aged 35 to 49; 4 years, 35 to 49=screening every four years for men aged 35 to 49; 3 years, 35 to 49=screening every three years for men aged 35 to 49; 2 years, 35 to 49=screening every two years for men aged 35 to 49; 1 year, 35 to 49=screening every one year for men aged 35–49; 5 years, ≥50=screening every five years for men aged ≥50; 4 years, ≥50=screening every four years for men aged ≥50; 3 years, ≥ 50=screening every three years for men aged ≥50; 2 years, ≥50=screening every two years for men aged ≥50; 1 year, ≥50=creening every year for men aged ≥50.

Figure 1 is the cost-effectiveness plane of the non-dominated strategies (connected lines) compared with dominated strategies (those that are found above the line). The cost-effectiveness plane plots each strategy to show the difference in effectiveness against the difference in cost. The strategies above the line are the dominated strategies (i.e. higher ICER in comparison with those connected by the line). Therefore, strategies that are connected by the line are the most cost-effective options (i.e. lowest ICER).

ICERs were most influenced by the cost of a false-positive result, the probability of detection outside a formal screening program, discount rate, the progression rate of anal cancer and specificity of DARE (Supplementary file 5). Results were less sensitive to costs of screening/workup/management and utility weights of local/regional/distal cancer. Table 4 provides a selected summary of the univariate sensitivity analyses to the model parameters most likely to vary.

**Table 4:** Sensitivity analyses of discounted ICER ($ per QALY gained) of three screening strategies for men aged ≥50

Variable	Base-case values	Sensitivity analysis values	Screening every two years (compared with every three years)	Screening every three years (compared with every four years)	Screening every 4 years (compared with every 5 years)
Cost of false-positive ($)	218				
		100	25,445	18,536	17,128
		500	93,134	65,605	57,626
Probability of detecting localized cancer without screening	0.2				
		0.1	40,475	29,023	26,172
		0.55	83,118	58,138	50,831
Specificity of DARE	0.25				
		0.1	52,791	37,552	33,489
		0.5	33,117	23,871	21,717
Discount rate	3%				
		0%	29,648	21,125	19,650
		5%	61,155	44,074	38,795
Progression rate	1 year				
		2 years	76,095	52,635	44,313
		3 years	118,443	79,514	65,267
Survival after treatment		5% better 5% worse	25,755 74,837	19,957 50,008	19,246 41,125

**Figure 2:**
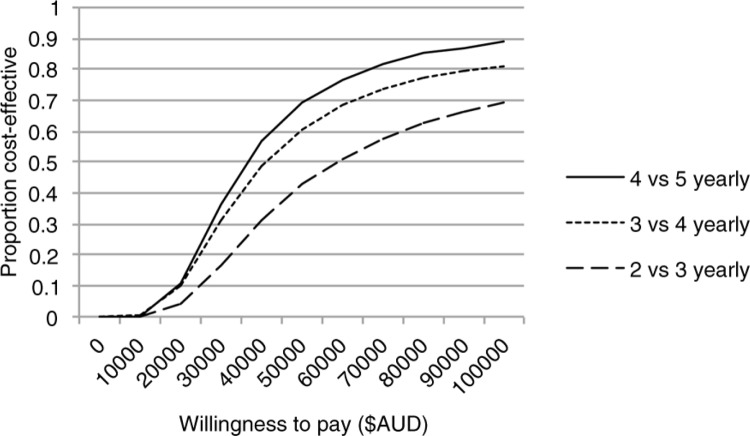
Cost-effectiveness acceptability curve from the probabilistic sensitivity of screening strategies for men aged >50.

We present the uncertainty in cost-effectiveness by means of a cost-effectiveness (Figure 2). This curve can be used to find the point estimate of cost-effectiveness and most importantly, a decision-maker who knows their maximum willingness to pay for health gain can use the curve to find the strength of evidence in support of the intervention being cost-effective. For example, if the willingness to pay was $50,000 per QALY gained, the likelihood of screening for men aged ≥50 every four years being cost-effective was 71%, every three years was 60% and every two years was 41%. If the willingness to pay was $100,000 per QALY gained, the likelihood of screening every four years for men aged ≥50 being cost-effective was 90%, every three years was 81% and every two years was 70%. The cost-effectiveness scatter plot with 95% confidence interval can be seen in Supplementary file 6.

## Discussion

To our knowledge, no cost-effectiveness studies have been conducted for evaluating the role of DARE in anal cancer screening. Our analyses suggest that regular anal screening MSM living with HIV aged ≥50 may be cost-effective, according to the frequently quoted accepted scale of willingness to pay (i.e. <$50,000 pre-QALY) [30]. Importantly, this is comparable with other cancer screening programmes such as cervical cancers [31]. As willingness to pay for a QALY can be variable and is related to the country's gross domestic product (GDP) per capita, our acceptability curve (Figure 2) provides information for policy makers depending on societal preferences. Our sensitivity analyses also assessed variations in the costs of management of anal cancer to levels consistent with those reported in the United Kingdom and the United States [5,6] but it did not change our final results.

Our results were sensitive to several factors. Firstly, the cost of false-positives (i.e. lesions that were referred but were not cancer) influenced the ICERs the most. We were conservative in our model, using the same rate of false-positives throughout the lifetime of screening but it is important to note that in reality, it is likely that repeat examinations may result in fewer referrals and improved specificity of DARE. In another study, for example, there were no referrals as a result of the examination after the initial examination^17^. This suggests that over time, as referral rates fall, so will the ICER.

Secondly, the ICER was sensitive to the probability of anal cancer being detected outside a formal screening program. If men become more aware that they are at increased risk of anal cancer and hence present to a medical practitioner when they have symptoms, they are likely to present with potential anal cancer, outside a formal screening programme. Improving anal cancer symptom awareness provides another avenue for improving morbidity and mortality from anal cancer but would raise the ICER of the screening programme. Thirdly, the discount rate affected the ICERs. The discount rate devalues the long-term health benefits over time and places more value on immediate benefits. Given that a screening programme's costs are constant over time but the benefits are primarily in the long term, our model showed that if the discount rate was low (i.e. societal preference valued long-term gains), implementation of a screening program would be more cost-effective. Fourthly, the natural history of anal cancer influenced the ICER. If progression rates were slower than the base-case, our model suggests that less frequent screening would be more cost-effective.

The strengths of the model are that it closely simulated what is currently known about anal cancer from the SEER database (e.g. age-distribution of anal cancer diagnosed, stage-specific survival post diagnosis) and lifetime risk for anal cancer [1]. By utilizing a widely accepted methodology (i.e. Markov modelling) [13], we were able to evaluate the effect of screening over the lifetime of the cohort. Unlike other cost-effectiveness studies of anal cytological screening, which estimated anal cancer management cost from those for colorectal cancer, we used the most up-to-date direct cost estimations for screening and management of anal cancer based on our clinical studies and the latest clinical guidelines for treatment of stage-specific anal cancer [10].

There are some limitations to our analyses. Due to lack of accurate data regarding the impact of comorbidities on utility weights for MSM living with HIV over time, our model's calculation of QALYs did not adjust for the loss of quality of life that would result from ageing and other comorbidities associated with HIV in the absence of cancer. This would make our model more accurate and complex but was beyond the scope of our analysis. We used several clinical parameters from a representative sample of MSM living with HIV [32] such as the proportion of patient uptake of screening, costs of screening (including extra consultation time, complication rates from screening) and utility weights for calculating QALYs. These parameters may differ according to other clinical settings, but we ensured that sensitivity analyses for these parameters were conducted to account for the uncertainties in these values used in the base-case scenario. We used SEER data to calculate the age-specific incidence rate and stage-specific survival from anal cancer. The SEER data only include US data and does not distinguish those who were MSM living with HIV. Having more accurate incidence and survival data for MSM living with HIV diagnosed in the various stages of anal cancer would improve the accuracy of the model. Our study also assumed standard treatment of chemoradiation would be received by those diagnosed with different stages of cancer other than those surgically removed (conservatively assumed to be 5% of localized cancers in our base-case). However, with the emergence of better treatments for anal cancer (e.g. intensity-modulated radiation therapy which may be better tolerated with higher survival rates [28]), implementing a screening programme that detects many more smaller tumours would result in less expensive treatments. The sensitivity analyses showed that screening would be more cost-effective even if current survival from treatment modalities increased by just 5% (Table 4). This means that the other two strategies (i.e. screening men aged ≥50 every year or every five years) may also be cost-effective and should be considered as a viable screening interval as survival from treatment modalities continue to improve over time. Furthermore, treatment costs would also be reduced if studies showed that very small anal canal tumours could also be treated by surgery alone. An additional benefit of regularly conducting DARE may be increased detection of rectal and prostate cancer but this was beyond the scope of our study to evaluate.

## Conclusions

This study modelled the cost-effectiveness of DARE because a clinical prospective study to compare the efficacy of implementing DARE for early anal cancer detection in MSM living with HIV is unlikely to be conducted (needing large numbers of men followed up for many years). Given that an annual DARE was feasible to be implemented into routine HIV care and that MSM living with HIV found regular DARE to be acceptable, simple and safe, with no major reported adverse effects, we recommend that regular anal examinations (every 1 to 5 years depending on the willingness to pay threshold) into routine HIV care for MSM aged ≥50 should be implemented as it is likely to be cost-effective. The cost-effectiveness of this recommendation would improve if we can reduce the costs of extra investigations by the specialist referral, improve the specificity of DARE (by upskilling HIV physicians to manage common anal conditions and only referring lesions suspicious for anal cancer) and improve survival from anal cancer treatments.

## Supplementary Material

Cost-effectiveness of screening for anal cancer using regular digital ano-rectal examinations in men who have sex with men living with HIV
