# Supplementary material for: Cost-effectiveness of screening for anal cancer using regular digital ano-rectal examinations in men who have sex with men living with HIV
Source: J Int AIDS Soc. 2016 Mar 1;19(1):20514. doi: 10.7448/IAS.19.1.20514 (PMC4778406; doi:10.7448/IAS.19.1.20514)

Appendix 1.1 Costs of screening for anal cancer

Component	Item number	Unit cost ($A)	Number of units	Cost	% of patients	Overall cost ($A)
5 minutes extra consulting time	MBS 23	37	0.25	9	100	9
0.3% of screened men returned for medical attention	MBS 23	37	1	37	0.3	0.1
Referred to colorectal surgeon	MBS 104	86	1	86	5	4
Examination under anaesthesia<br>Biopsy<br>Colorectal surgeon – subsequent visit	MBS 20902<br>MBS 30071<br>MBS 105	79<br>52<br>43	1<br>1<br>1	79<br>52<br>43	2<br>2<br>2	1<br>1<br>1
Total						16

Appendix 1.2 Costs for workup of anal cancer

Component	Item number	Unit cost ($A)	Number of units	Cost ($A)	% of patients	Overall cost ($A)
Colorectal surgeon – initial visit	MBS 104	86	1	86	100	86
Colorectal surgeon – subsequent visit	MBS 105	43	1	43	100	86
Sigmoidoscopy	MBS 32072	48	1	48	100	48
Examination under anaesthesia	MBS 20902	79	1	79	100	79
Biopsy	MBS 30071	52	1	52	100	52
CT chest/abdomen/pelvis	MBS 56807	560	1	560	100	560
PET scan	MBS 61541	953	1	953	100	953
Total						1864

Appendix 1.3 Costs for false positive from screening

Component	Item number	Unit cost ($A)	Number of units	Cost ($A)	% of patients	Overall cost ($A)
Colorectal surgeon – initial visit	MBS 104	86	1	86	100	86
Colorectal surgeon – subsequent visit	MBS 105	43	1	43	100	86
Examination under anaesthesia	MBS 20902	79	1	79	35	28
Biopsy	MBS 30071	52	1	52	35	18
Total						218

Appendix 1.4 Costs for monitoring patient with anal cancer

Component	Item number	Unit cost ($A)	Number of units	Cost ($A)	% of patients	Overall cost ($A)
Year 1						
Radiation oncologist weekly for 4 weeks, then every 3 months	MBS 105	43	7	301	100	301
Colorectal surgeon review every 3 months	MBS 105	43	4	172	100	172
CT chest/abdomen/pelvis	MBS 56807	560	1	560	50	280
Year 2						
Radiation oncologist review every 6 months	MBS 105	43	2	86	100	86
Colorectal surgeon review every 6 months	MBS 105	43	2	86	100	86
CT chest/abdomen/pelvis	MBS 56807	560	1	560	50	280
Year 3						
Colorectal surgeon review every 6 months	MBS 105	43	2	86	100	86
CT chest/abdomen/pelvis	MBS 56807	560	1	560	50	280
Colonoscopy	ARDRG G48C	1935	1	1935	100	1935
Year 4 +5						
Colorectal surgeon review every 6 months	MBS 105	43	2	86	100	86
Year 6						
Colorectal surgeon review annually	MBS 105	43	1	43	100	43
Colonoscopy	ARDRG G48C	1935	1	1935	100	1935
Year 7+8						
Colorectal surgeon review annually	MBS 105	43	1	43	100	43
Year 9 and beyond: 3 year cycles where a colorectal surgeon will review annually and every 3rd year a colonoscopy is done						

Appendix 1.5 Costs for cancer treatment

Component	Item number	Unit cost ($A)	Number of units	Cost ($A)	% of patients	Overall cost ($A)
Local cancer treatment						
Colorectal surgeon consultation	MBS 105	43	1	43	10	4
Curative excision	ARDRG G11Z	3998	1	3998	10	400
Radiation oncologist – initial visit	MBS 104	86	1	86	90	77
Radiation oncologist – subsequent visit	MBS 105	43	1	43	90	39
Radiotherapy – 6 weeks	-	9700	1	9700	90	8730
Medical oncologist – initial visit	MBS 104	86	1	86	90	77
Medical oncologist – subsequent visit	MBS 105	43	2	86	90	77
Full blood examination	MBS 65070	17	1	17	90	15
Urea and electrolytes	MBS 66509	16	1	16	90	14
Liver function test	MBS 66512	18	1	18	90	16
5 fluorouracil (1000mg)	PBS 4431F	108	8	864	90	778
Mitomycin (10 mg)	-	177	1	177	90	159
**Total local cancer treatment**						10,386
Regional cancer treatment						
Radiation oncologist – initial visit	MBS 104	86	1	86	100	86
Radiation oncologist – subsequent visit	MBS 105	43	1	43	100	43
Radiotherapy for 6 weeks	MBS 15550, 15562, 15705/15710 (X13), 15254 (X36), 15269.005 (X36)	9700	1	9700	100	9700
Medical oncologist – initial visit	MBS 104	86	1	86	100	86
Medical oncologist – subsequent visit	MBS 105	43	2	86	100	86
Full blood examination	MBS 65070	17	1	17	100	17
Urea and electrolytes	MBS 66509	16	1	16	100	16
Liver function test	MBS 66512	18	1	18	100	18

5 fluorouracil (1000mg)	PBS 4431F	108	8	864	100	864
Mitomycin (10 mg)	-	177	1	177	100	177
**Total regional cancer treatment**						11,093
Distal cancer treatment						
Radiation oncologist – initial visit	MBS 104	86	1	86	100	86
Radiation oncologist – subsequent visit	MBS 105	43	1	43	100	43
Radiotherapy (limited)	-	1000	1	1000	50	500
Medical oncologist – initial visit	MBS 104	86	1	86	100	86
Medical oncologist – subsequent visit	MBS 105	43	2	86	100	86
Cisplatin (100 mg)	PBS 4319H	130	6	780	100	780
5 fluorouracil (1000 mg)	PBS 4431F	108	30	3240	100	3240
Full blood examination	MBS 65070	17	1	17	100	17
Urea and electrolytes	MBS 66509	16	1	16	100	16
Liver function test	MBS 66512	18	1	18	100	18
Palliative care physician – initial visit	MBS 104	86	1	86	100	86
Terminal care	Personal communication[1]	9680	1	9680	100	9,680
**Total distal cancer treatment**						14,638

[1] Personal communication with Lynne Conway (re: CSL Biotherapies PBAC submission for Gardasil). An estimated cost of terminal care was provided in the submission by Prof. Kathy Eager.

Appendix 2 Estimated anal cancer incidence by age in men who have sex with men living with HIV (base-case analysis) compared with anal cancer rates in the US general population[27]

Using Seer Database to calculate anal cancers in men 1973 to 2011 database.

Age	Rate per 100,000 (General population)	Count	Population	Estimated rate per 100,000 (MSM living with HIV)*
35-39	0.7	251	36,315,640	17.85
40-44	1.4	516	37,276,063	35.7
45-49	1.9	689	36,209,959	48.45
50-54	2.2	728	32,731,724	56.1
55-59	2.6	691	27,085,324	66.3
60-64	2.7	556	20,877,075	68.85
65-69	3.2	495	15,484,756	81.6
70-74	3.3	397	12,130,131	84.15
75-79	3.7	350	9,564,755	94.35
80-84	3.6	241	6,613,363	91.8
85+ years	3.9	187	4,794,246	99.45

* Assuming anal cancer incidence was 45.9 per 100,000 in MSM living with HIV and 1.8 per 100,000 for males in the general population[27], we multiplied the rates in the general population by (45.9/1.8) to estimate the rates in MSM living with HIV.

Appendix 3 Survival after diagnosis of anal cancer according to stages.

Survival proportion according to SEER data[25]

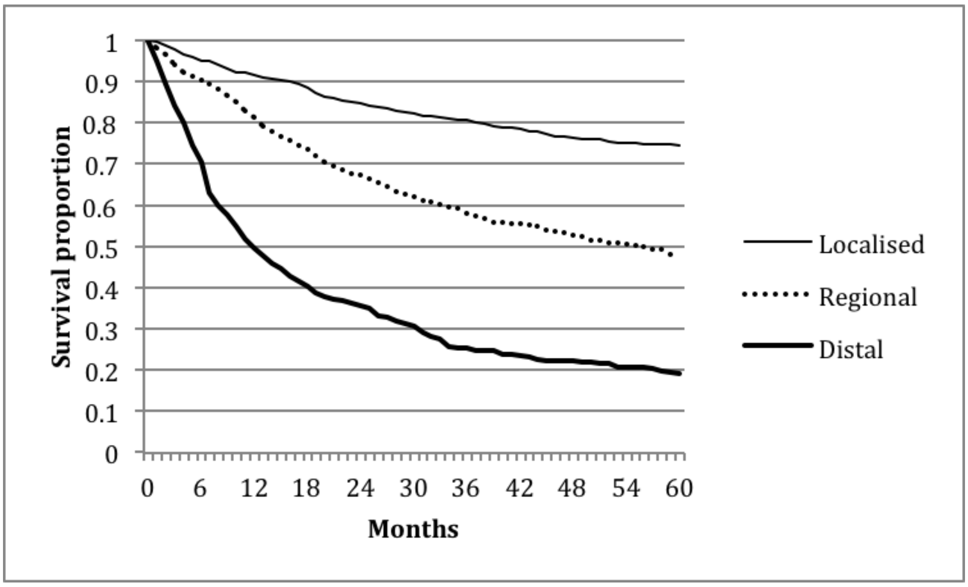

Survival proportion estimated from Weibull equations

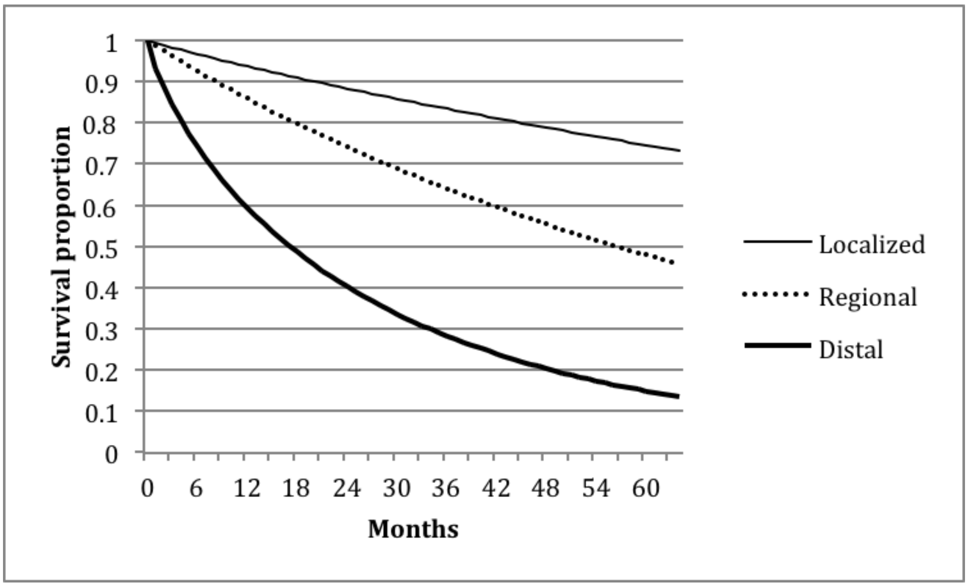

Localised cancer: EXP(-EXP(-5.060296)*t_LocalCancerRemission^0.9363879)

Regional cancer: EXP(-EXP(-4.311012)*t_RegCaRemission^0.977784)

Distal cancer: EXP(-EXP(-2.651782)*t_DistCaRemission^0.8052466)

Appendix 4 Tornado diagram of incremental cost-effectiveness ratio of 4 yearly screening of men aged > 50 years compared with no screening

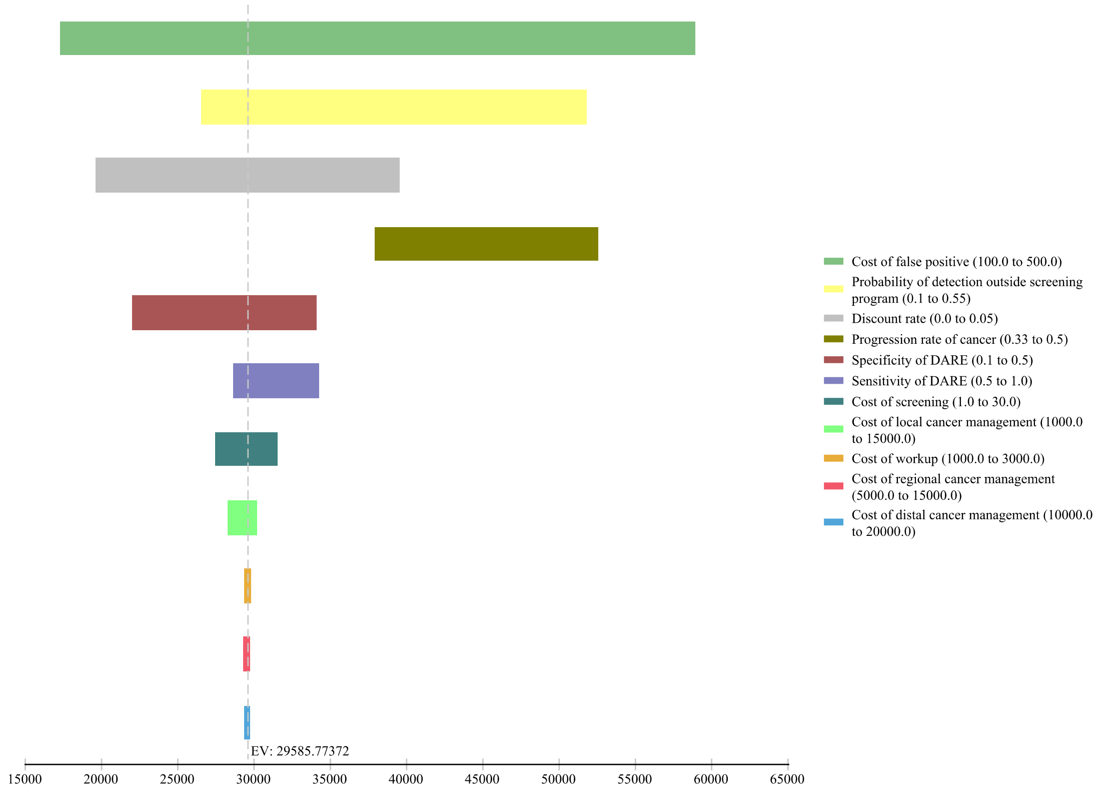

Supplement: Cost-effectiveness of screening for anal cancer using regular digital ano-rectal examinations in men who have sex with men living with HIV [file JIAS-19-20514-s001.pdf]
